# Biochemical methane potential of microalgae biomass using different microbial inocula

**DOI:** 10.1186/s13068-018-1188-7

**Published:** 2018-06-29

**Authors:** Cristina Gonzalez-Fernandez, Santiago Barreiro-Vescovo, Ignacio de Godos, Maikel Fernandez, Arbib Zouhayr, Mercedes Ballesteros

**Affiliations:** 1 Biotechnological Processes Unit, IMDEA Energy, Madrid, Spain; 2 Aqualia Gestión Integral del Agua SA, Madrid, Spain; 3 Biofuels Unit, CIEMAT, Madrid, Spain

**Keywords:** Microalgae, Anaerobic digestion, Anaerobic microbiome, Specific methanogenic activity, Inoculum

## Abstract

**Background:**

Microalgae biomass is regarded as a potential feedstock for bioenergy purposes through anaerobic digestion (AD). Even though AD is a well-proven technology, the use of new feedstocks requires in-depth studies. A lot of research has been conducted assessing methane yield without paying attention to the anaerobic microbiome and their activities. For such a goal, the present investigation was designed to link methane yield to those two later sludge characteristics. In this sense, different anaerobic sources were tested, namely adapted to microalgae biomass and adapted to sewage sludge.

**Results:**

Despite the registered differences for the anaerobic microbiome analysis and specific methane activities towards model substrates, sludge adapted to digest sewage sludge did not affect the methane yield of *Chlorella sorokiniana* and *Scenedesmus* sp. Opposite to that, sludge samples adapted to digest microalgae exhibited a concomitant increase in methane yield together with increasing digestion temperatures. More specifically, the values attained were 63.4 ± 1.5, 79.2 ± 3.1 and 108.2 ± 1.9 mL CH_4_ g COD in^−1^ for psychrophilic, mesophilic and thermophilic digestions, respectively. While psycro- and mesophilic digestion supported similar yields (most probably linked to their anaerobic microbiome resemblance), the values attained for thermophilic digestion evidenced the usefulness of having a highly specific microbiome. The relative abundance of Firmicutes, particularly *Clostridia*, and Proteobacteria together with an important abundance of hydrogenotrophic methanogens was highlighted in this inoculum.

**Conclusion:**

Overall, this study showed that working with tailored anaerobic microbiome could help avoiding pretreatments devoted to methane yield enhancement.

## Background

Renewable energy will play a key role in limiting CO_2_ emissions while decreasing as well fossil fuel dependency. Renewable resources such as biomass and organic wastes are envisaged as promising feedstock for energy purposes. Anaerobic digestion is a proven technology for obtaining biogas via conversion of organic matter. In this regard, anaerobic digestion (AD) has been implemented in a large number of facilities and in fact, this number increases over the years [1]. Facilities already running use mainly traditional feedstocks such as manure or energy crops (corn). However, the need of expanding AD to a range of new substrates has raised attention in key points that should be taken into account when new feedstocks are going to be used. Microalgae biomass has been the focus of interest in AD during the last decade. Microalgae biomass is a substrate of particular interest since it contributes to wastewater bioremediation and CO_2_ bio-mitigation. Additionally, microalgae biomass can be grown in non-arable land and are more productive than terrestrial plant due to their higher photosynthetic efficiency [2]. All these features render microalgae biomass as a promising feedstock for energy purposes.

Out of the different energy forms that can be produced using microalgae biomass as feedstock, biogas production via anaerobic digestion is probably the most economically feasible since it does not require highly concentrated biomass [3] and anaerobes can use the three biomass macromolecules (proteins, carbohydrates and lipids) for methane production purposes [4]. As a limiting step, anaerobic hydrolysis appears to be one of the most challenging steps to reach a positive economic balance and to completely exploit the potential of microalgae for biogas. Once evidenced the resilience of easily growing microalgae strains towards AD, a great number of investigations have been devoted to pretreat this biomass prior to anaerobic digestion. A summary of the research efforts that have been undertaken to date can be found elsewhere [5, 6]. Several authors have found that the energy consumption associated to the pretreatment of microalgae biomass is equal to or higher than the energy gained [7, 8].

One alternative to the use of pretreatments is the selection of appropriate anaerobic inocula. In fact, it has been shown that the use of one or another inoculum influences the biochemical methane potential of different substrates [9–11]. A consortium of interdependent microorganisms composes the anaerobic inoculum. The prevalence of determined anaerobes might affect the hydrolytic and methanogenic activity of the sludge and thus, different microbial population might lead to different anaerobic digestion efficiencies. Moreover, not only the inherent microbial activity of the sludge might be of importance but this activity can also be tuned by microbial adaptation. Anaerobic microbial community has the ability to adapt to changing conditions. Microbial adaptation to the new situation entails most of the time a lower microorganism diversity but more specified [12]. In this manner, some of the adaptation mechanisms include genetic changes, selective population enrichment [13] and enzymatic activity modification [14].

Although a number of studies have addressed the limited anaerobic biodegradability of this biomass and potential pretreatment methods to enhance it, controversial results might be found in the literature dealing with microalgae biomass digestion. One of the reasons of different results has been attributed to the different anaerobic sludge inocula used in the digestions. In this sense, to the best of our knowledge, no investigation has been made on the effect of different anaerobic inocula in the methane yield achievable when using microalgae as substrates. As a matter of fact, when comparing different inocula, studies mostly focus on methane production or substrate degradation while inocula themselves are not paid enough attention. For this reason, the aim of this investigation was to evaluate the effect of different inoculum sources on microalgae biomass anaerobic digestion. Six different inocula were tested in terms of anaerobic microbiome, degradation activity towards model substrates and methane production yield using microalgae as feedstocks. Those inocula included four operating at mesophilic range (35 °C), one psychrophilic (ambient temperature, 15–25 °C) and one thermophilic (55 °C). Moreover, the psychro-, thermo- and one of the mesophilic sludge were adapted to digest microalgae biomass while the rest of them were adapted to sewage sludge degradation.

## Methods

### Microalgae biomass used as substrate

The substrate consisted in two types of microalgae biomass, namely *Scenedesmus* gender and the specie *Chlorella sorokiniana. Scenedesmus* inoculum was grown in synthetic media in 1 L flask under continuous artificial light supply (TL-D 36W, PHILIPS, Holland). Cultures were kept at room temperature (22–24 °C) with magnetic stirring. Culture media consisted in (all compounds in mg L^−1^): 160 NH_4_Cl, 25 CaCl_2_·2H_2_O, 75 MgSO_4_·7H_2_O, 75 K_2_HPO_4_, 175 KH_2_PO_4_, 25 NaCl, 50 EDTA disodium, 31 KOH, 4.98 FeSO_4_·7H_2_O, 11.42 H_3_BO_3_, 17.64 ZnSO_4_·7H2O, 2.88 MnCl_2_·4H_2_O, 1.42 MoO_3_, 3.14 CuSO_4_·5H_2_O, and 0.98 CoNO_3_·6H_2_O.

*Scenedesmus* sp. was grown in a 200 L raceway reactor. The raceway was operated without temperature control (20–24 °C) and the illumination consisted in 12 fluorescent lamps. Culture was conducted in semicontinuous condition; harvested and fed once per week. Fresh urban wastewater from Rey Juan Carlos University was used as culture media for *Scenedesmus* sp. The specific composition of the influent consisted in (all elements in mg L^−1^): chemical oxygen demand (COD); total fraction 498 ± 190 (out of which 58% belongs to soluble fraction), N–NH_4_^+^ of 34.1 ± 16.3, N–NO_2_^−^ of 0.9 ± 0.8, N–NO_3_^−^ of 1.2 ± 1.1 and P–PO_4_^3−^ of 4.9 ± 3.6 and total suspended solids (TSS) and volatile suspended solids (VSS) of 0.17 ± 0.06 and 0.15 ± 0.04 g L^−1^, respectively. *Scenedesmus* sp. biomass was harvested with an industrial centrifuge (OTC3-02-137, 10,000 rpm, WESTFALIA).

On the other hand, *C. sorokiniana* was grown in synthetic media (previously described) in 1 L flasks for biochemical methane potential assay. Previously to the AD, this biomass was concentrated with the centrifuge MegaFUGE 16R (Thermo Scientific, EEUU) at 5000 rpm during 15 min. Second, *C. sorokiniana* biomass was frozen to compare the biogas production using a more easily degradable biomass than *Scenedesmus* sp. [15].

### Anaerobic digestion

#### Inocula: anaerobic sludge

The sludge samples (Table 1) were classified as mesophilic (35 °C) non-adapted to microalgae digestion and sludge samples adapted to microalgae digestion. Non-adapted anaerobic sludge samples were collected at the wastewater treatment plant of Valladolid (Valladolid, Spain, S1), Chiclana de La Frontera (Cádiz, Spain, S2), and La Reguera (Móstoles, Spain, S3). On the other hand, sludge samples adapted to microalgae digestion were collected in Chiclana de la Frontera (Cadiz, Spain). Those sludge samples were adapted to work at different operational temperatures, namely psychrophilic sludge (15–25 °C, S5), mesophilic (35 °C, S4) and thermophilic (50 °C, S6). Therefore, from now on, since the objective of this investigation is the AD of microalgae biomass, S4, S5 and S6 will be considered “adapted sludge”.

**Table 1:** Characteristics and chemical properties of sludge samples (S1–S6) employed as inoculum for anaerobic digestion

Parameter	S1	S2	S3	S4	S5	S6
Location	Valladolid	Chiclana	La Reguera	Chiclana	Chiclana	Chiclana
Substrate adaptation	Activated sludge	Activated sludge	Activated sludge	Microalgae	Microalgae	Microalgae
Temperature adaptation	Meso-	Meso-	Meso-	Meso-	Psyc-	Therm-
VS/TS	0.64	0.64	0.58	0.71	0.77	0.69
VSS/TSS	0.78	0.82	0.76	0.76	0.79	0.87
VSS/TS	0.56	0.62	0.58	0.47	0.51	0.60

#### Substrates: model compounds and microalgae biomass

Bovine serum albumin (BSA) as protein substrate and cellulose as carbohydrate substrate were chosen as model compounds for assessing the specific methanogenic activity (SMA, g COD consumed/g VSS day) of the different anaerobic inocula. These substrates (proteins and carbohydrates) were selected based on their prevalence in microalgae biomass grown in wastewater [16]. These SMAs were used as an indicator of proteolytic and cellulolytic activity of anaerobic sludge samples. Second, all inocula were employed to digest *Scenedesmus* sp. (fresh biomass and poorly biodegradable) and *C. sorokiniana* (previously frozen and thus, easily biodegradable biomass). The fresh *Scenedesmus* sp. biomass was stored at 5 °C during less than 5 days to preserve its physiological characteristics.

#### Biochemical methane potential (BMP)

The anaerobic digestion was carried out in enclosed reactors in batch mode during approximately 20–30 reaction days. BMPs were considered finished when cumulative methane production was stable. Digestion bottles had a total volume of 0.12 L and were incubated at different temperatures depending on the inoculum. Temperatures were set at room temperature (22–24 °C) for the psychrophilic assay (S5), at 35 °C for the mesophilic range (S1–S4) and at 50 °C for the thermophilic digestion (S6). Agitation was provided with an orbital shaker at 150 rpm. To ensure anaerobic conditions, bottles were purged with helium, closed with butyl rubber and aluminum caps. Calculations were set to achieve a final working volume of 0.07 L, allowing a head space of 42% for biogas production. The inocula and anaerobic sludge samples were mixed with the different model substrates and algae biomass in a ratio of COD_substrate_/VS_inoculum_ of 0.5 (g g^−1^). Biodegradability assays were carried out in triplicates. The methane volume generated was calculated with the pressures determined in the head space bottle. The net methane production at standard temperature and pressure (STP) was calculated by subtracting the blank methane production to the amount of methane measured in each sample. To determine the endogenous methane production, bottles containing only anaerobic sludge samples were run as a blank.

### Analytical methods

Total solids (TS), volatile solids (VS), total suspended solids (TSS), volatile suspended solids (VSS) and the total Kjeldahl nitrogen (TKN) were measured according to standard methods [17]. Total proteins were calculated by multiplying TKN values by 5.95. Carbohydrates content were measured employing phenol sulphuric [18]. Colorimetric commercial methods were used for quantification of COD and ammonium (Merck, ISO 15705 y ISO 7150-1, respectively). N–NO_2_^−^, N–NO_3_^−^, and P–PO_4_^3−^ were determined by ion chromatography (IC 9030, METHROM, Switzerland). Soluble fractions were obtained by centrifuging at 5000 rpm for 15 min with the centrifuge MegaFUGE 16R (Thermo Scientific, USA) and later filtering by nylon filter of 0.45 µm porous diameter. Biogas composition was determined by gas chromatography (Agilent 7820A) equipped with a 30 m column (HP-PLOTQ, 0.53 mm, 40 µm) connected to a thermal conductivity detector at 250 °C and helium flow of 4.5 mL min^−1^ as gas carrier.

### Inocula microbial community analysis: pyrosequencing

Total DNA extraction of anaerobic sludge samples was performed using FastDNA Spin kit for soil (MPBiomedicals, LLC). Before DNA amplification reaction, samples were purified using commercial kit QiAmp DNA Microkit (QIAGEN, USA). Library preparation was made using set primer based on the capturing of 16s rRNA region as described in Klindworth et al. [19]. Sequencing samples were loaded in the MiSeq Platform from Illumina using a 300PE combination. Sequences were compared against the built rRNA database using a BLAST local alignment approach to associate each cluster to one taxonomical group from the database. Pyrosequencing was performed by Lifesequencing S.L. (University of Valencia, Spain).

### Statistical analysis

Data were presented as means values ± standard deviation of the mean and statistical significances were assessed by analysis of variance (ANOVA). Values were considered statistically significant when *p* value was lower than 0.05.

## Results and discussion

### Sludge samples and microalgae chemical characteristics

All anaerobic sludge samples exhibited a standard VS/TS ratio ranging 0.66 ± 0.05, VSS/TSS = 0.80 ± 0.04 and VSS/VS = 0.57 ± 0.07. These values were in good agreement with other sludge samples used for BMP tests [20, 21]. In this manner, despite the different feedstock used during their previous activity, namely sewage sludge (mixture of primary and secondary sludge) (S1, S2 and S3) or microalgae biomass (S4, S5 and S6), all of them were within the usual values.

With regard to the microalgae biomass employed as substrate, both biomass showed a prevalent protein composition (Table 2). This feature is quite normal of microalgae grown without any stressful condition [20]. In the case of *Scenedesmus* sp., proteins were followed by carbohydrates (approx. 34%) and a minor proportion of lipids (11%). Opposite to that, *C. sorokiniana* displayed a higher lipid content (24%) than the carbohydrate fraction (10%). It should be noted that this strain has been reported to have a particular tendency to accumulate lipids [22, 23].

**Table 2:** Macromolecular distribution of the microalgae biomass employed as feedstock for anaerobic digestion

Chemical parameter	*Scenedesmus* sp.	*C. sorokiniana*
TS g L^−1^	26.7 ± 0.7	37.5 ± 0.2
VS g L^−1^	23.0 ± 1.2	34.1 ± 0.0
Carbohydrates (%)	33.8 ± 2.4	10.4 ± 0.7
Proteins (%)	41.0 ± 0.7	56.3 ± 0.7
Lipids (%)	11.5 ± 3.4	24.1 ± 0.7
Ash (%)	13.8 ± 2.5	9.2 ± 0.3

### Sludge samples: anaerobic microbiome

Pyrosequencing was performed to characterize the anaerobic microbiome of all sludge samples. With regard to Shannon’s index of diversity at genera level, all the sludge samples were in the range of 1.36–1.75, exception made for S1 and S6, which exhibited 3.07 and 0.32, respectively. This indicated that the diversity of S1 was considerably higher than the rest of sludge samples while S6 displayed a highly specific consortium in which the biodiversity was really reduced.

**Fig. 1:**
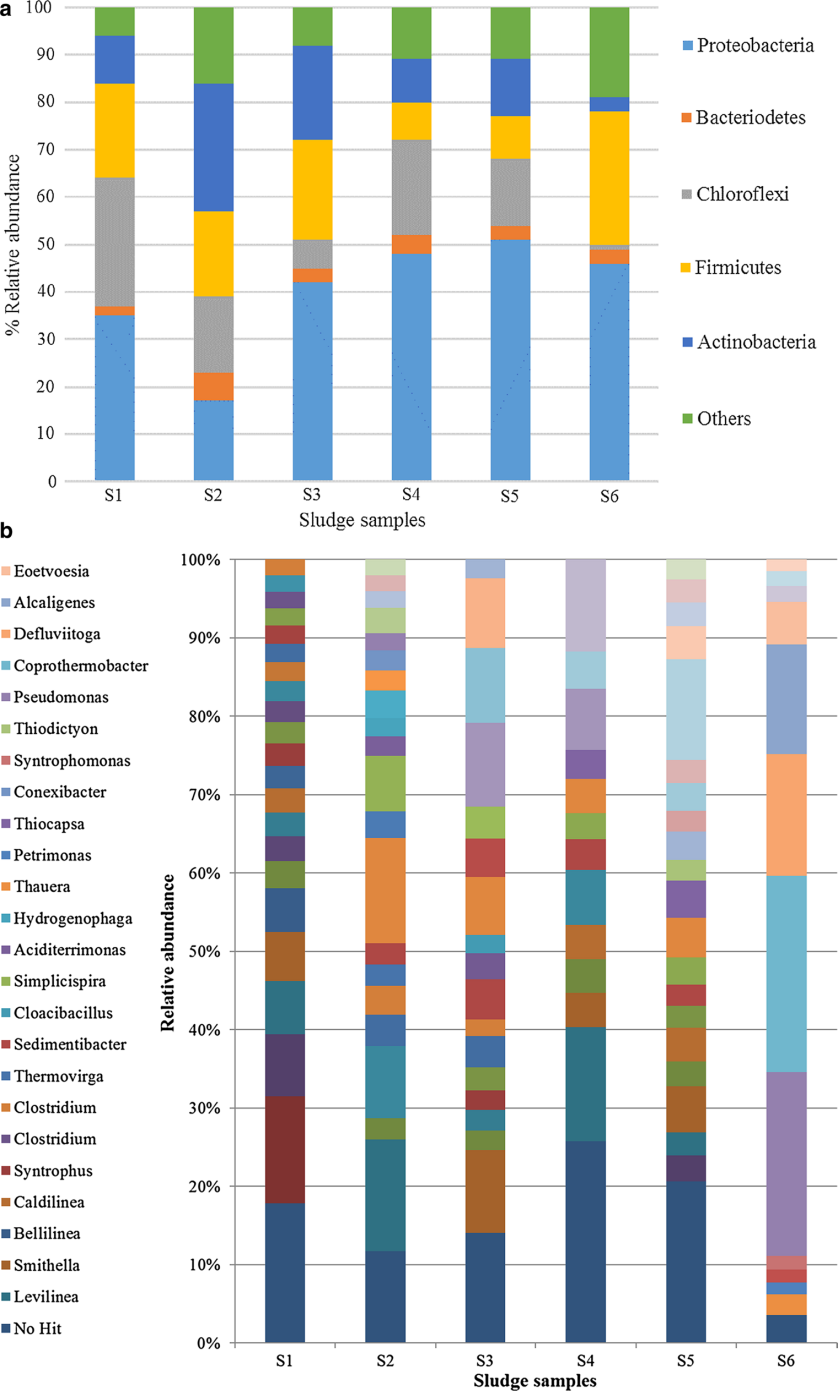
Taxonomic profiles at phylum (**a**) and genera (**b**) level for the bacterial community of the different inoculum sources used in the BMPs

At phylum level, bacterial distribution was mainly represented by Proteobacteria. This phylum ranged 46–51% of the bacteria retrieved in the adapted sludge samples while in S2, the prevalence of Proteobacteria was outcompeted by Actinobacteria (27%) and S1 and S3 exhibited 35 and 42% of the bacterial population (Fig. 1a). Proteobacteria are frequently reported to be present at high proportion in anaerobic sludge [24]. Groups of Proteobacteria as *Rhizobiales, Rhodobacterales, Sphingomonadales* and *Burkholderiaels*–*Comamonadaceae* found in this work have been also reported in studies related to microalgae-based wastewater treatment [25]. Reactors S3–6 were adapted to digest microalgae–bacteria consortia harvested from a photosynthetic-based wastewater treatment. This inocula adaptation can explain the prevalent presence of this group of bacteria in these inocula. It has been shown that Proteobacteria population increases from 13 to 50% when changing the feeding from sewage sludge to raw *Chlorella* [26]. This fact was also observed in the present investigation, in which the sludge samples adapted to digest microalgae (S4–S6) exhibited slightly higher population of this phylum. This feature could be explained by the above-mentioned prevalence of proteins in microalgae biomass.

The second main phyla retrieved from the sludge samples was Firmicutes. Non-adapted sludge (S1–S3) showed a relative abundance of approximately 20% of Firmicutes (Fig. 1a). This bacterial community is also normally present in anaerobic sludge digesting sewage sludge. Interestingly, adapted sludge samples (S4–S6) decreased their abundance of Firmicutes in psychrophilic and mesophilic sludge to around 10% while that of the thermophilic displayed 28% relative abundance. The sequences were mainly affiliated to the order *Clostridiales*. More specifically, the relative abundance of *Clostridia* in S2–S5 were in the narrow range of 62–66% of the Firmicutes, while S1 and S6 showed markedly higher percentages (86 and 96%, Fig. 1b). It is important to note that *Clostridia* are responsible of conducting macromolecules hydrolysis [27] and syntrophic acetate oxidation coupled with hydrogenotrophic methanogenesis [28]. *Clostridiales* is an order of obligate anaerobic bacteria with chemoorganotrophic fermentative metabolisms. Members of this group of microorganisms are frequent in soils, sediments, rumen and intestinal tracts of animals and insects. In addition, *Clostridiaceae* family members have in common their saccharolytic fermentative metabolism in environmental substrate decomposition [29].

Bacteroidetes were present at really low relative abundance (2–6% for all sludge samples). Bacteroidetes are anaerobic microorganisms which are included in animal intestinal microflora [30] due to its importance in cellulose and protein degradation [31]. When digesting raw and thermally pretreated *Chlorella*, Bacteroidetes abundancy increased to 20% with regard to 12% registered in the inoculum used as seed of the reactor (adapted to digest sewage sludge, [26]). Likewise, Bacteroidetes also found the dominant phylum in the digestion of *Scenedesmus obliquus* at mesophilic range [32]. Nevertheless, in the present study, their abundance remained low regardless of the inoculum source.

Chloroflexi phylum are aerobic bacteria commonly found in activated sludge systems [33], and therefore, this phylum usually ends up in the anaerobic digesters of wastewater treatment plants. Due to their filamentous morphology, their presence is normally associated to bulking phenomena. In this manner, no trend could be withdrawn in sludge samples collected from wastewater treatment and their relative abundance varied from 6% for S3 to 27% for S1. A striking feature was the fact that the thermophilic adapted sludge showed 1% of this phylum (Fig. 1a). This value was really low when compared to the rest of sludge samples. Nevertheless, this feature was in agreement with Greses et al. [34] who also reported extremely low relative abundance (1.1%) in a thermophilic CSTR fed with S*cenedesmus*. Authors attributed this fact to the low-ammonium tolerance of this phyla and the operational temperature. It should be also highlighted that S6 displayed 16% *Thermotogae* relative abundance (classified as other in Fig. 1a). The presence of this phylum was negligible in the rest of the sludge samples. *Thermotogae* have been described to release hydrolytic enzymes catalyzing the degradation of polysaccharides into acetate, carbon dioxide and hydrogen [35] and also to play a key role in interspecies hydrogen transfer [36]. *Thermotogae* species are obligate anaerobes and hyperthermophiles bacteria found in large range extremophile environments, including mammalian, ruminant and termite digestive tracts. All those ecophysiology characteristics explain this abundance in S6 sludge [37].

**Fig. 2:**
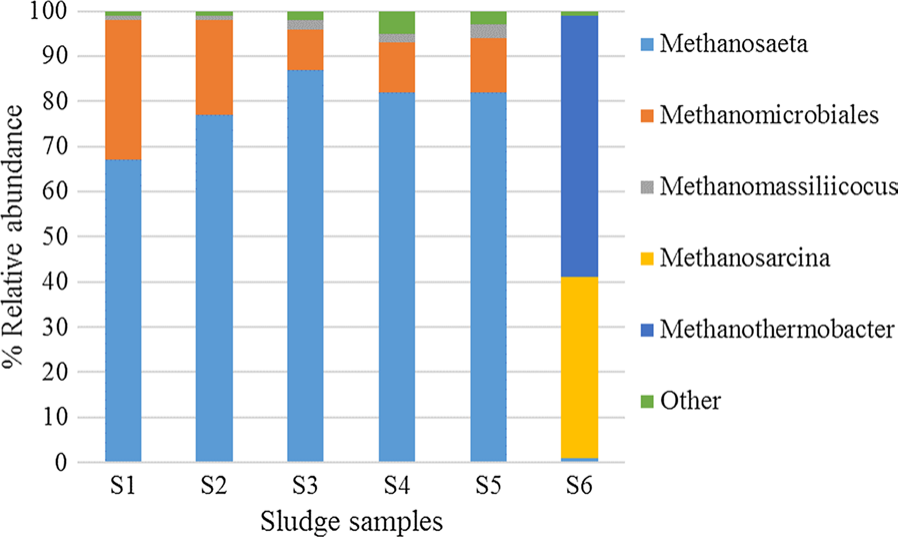
Taxonomic profiles at phylum level for the archaeal community of the different inoculum sources used in the BMPs

The archaeal domain accounted for 7–8% of the identified population in the case of sludge adapted to digest sewage sludge (S1–S3), while this value decreased to 3–4% for all the sludge samples adapted to digest microalgae (S4–S6). In this manner, it was clear that microalgae digestion affected archaeal relative abundance. With regard to the phyla determined in the different sludge samples, the abundance of the strict acetotrophic *Methanosaeta* was prevailing in S1–S5, ranging from 67 to 82% of the archaeal population (Fig. 2). Opposite to that, methanogenesis in the thermophilic sludge S6 was mainly conducted by *Methanothermobacter* and *Methanosarcina* (59 and 40% of the archaeal population retrieved in the sample, respectively). *Methanothermobacter* is a hydrogenotrophic methanogen [38] while *Methanosarcina* is more versatile and can metabolize both hydrogen and acetate as energy source [39]. Indeed, acetoclastic methanogens are commonly outcompeted by hydrogenotrophic methanogens in thermophilic digesters [40]. This fact is related to the lower stability of thermophilic digestion conditions as a result of acetate accumulation and acidification. In this manner, according to the bacterial and archaeal population it can be assumed that this was the case as well in S6. Hydrogenotrophic methanogens were also represented by the *Methanomicrobiales* community identified in S1–S5, however, their relative abundance was much lower than in the thermophilic digester (10–30% vs. 60%).

There are no other studies published that focused in the comparison of the microbial population inocula adapted to digest microalgae and sewage sludge at different temperatures ranges. As an example, many other articles have characterized the microbial composition of inocula adapted to digest microalgae and activated sludge only at one temperature range [24, 25, 29, 34]. The novelty of this article is the comparison of different microbiome composition adapted to three different operational temperature ranges and feedstocks (microalgae and raw sewage). Overall, the anaerobic microbiome analysis of all sludge samples used in the present study evidenced different bacterial and archaeal population not only in terms of relative abundances but also in the phyla and genera identified. In this manner, it could be concluded that the different feedstocks fed in the digesters (sewage sludge and microalgae biomass) as well as the operational temperature had an impact on the inoculum sources.

### Specific methanogenic activity of sludge samples toward model substrates: BSA and cellulose

**Fig. 3:**
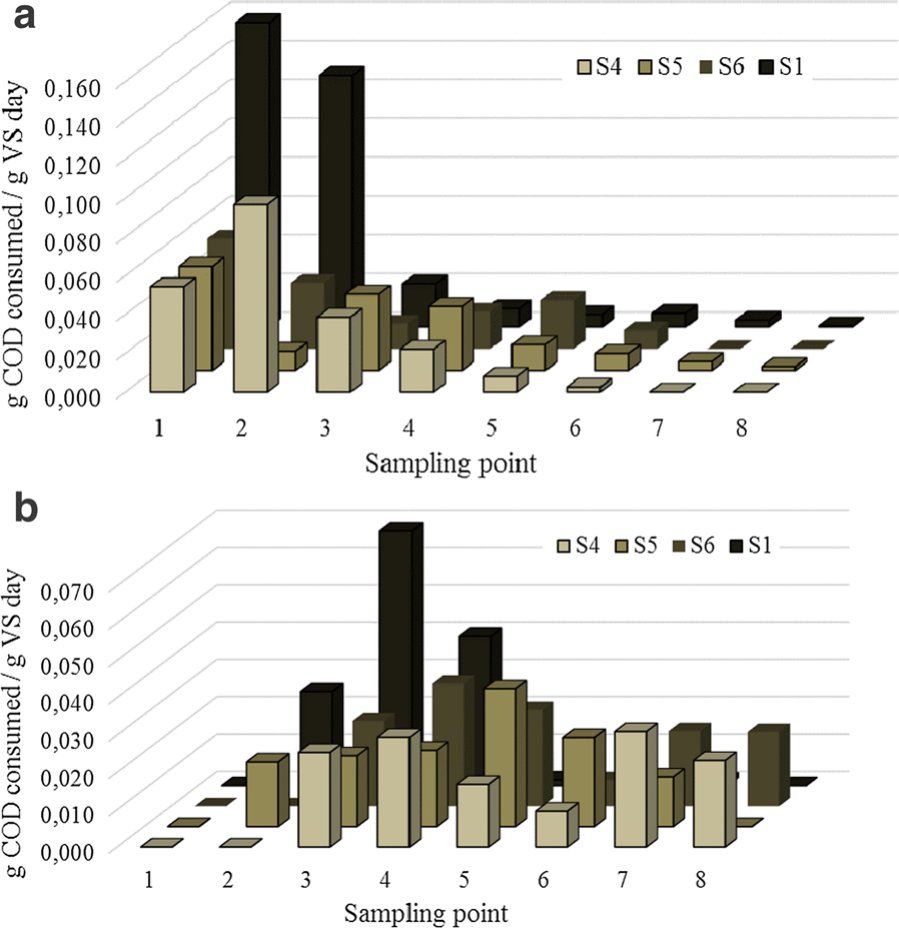
Specific methanogenic activities (SMA) towards model substrates: **a** BSA and **b** cellulose

According to Angelidaki et al. [41] specific methanogenic activity (SMA) was measured using different model substrates representing proteins (BSA) and carbohydrates (cellulose). Those two substrates were selected as model for protein and carbohydrate degradation since those are the fractions which present lower biodegradability in the microalgae biomass [16, 20]. As it can be seen in Fig. 3, SMA was higher and faster for the BSA model substrate than with cellulose. The sludge S1 exhibited considerably higher SMA than the adapted sludge samples. More specifically, in the first day of digestion, S1 displayed threefold SMA (0.157 g COD consumed/g VS day) than S4, S5 and S6 (0.055 g COD consumed/g VS day, Fig. 3a). This fact was probably mediated by the higher microbial diversity encountered in this sludge in comparison to the adapted ones (Section “Sludge samples: anaerobic microbiome”). A higher diversity implies more options that the appropriate degrading microorganisms is within the microbial consortium, which ultimately implies more degrading pathways that are strictly required for anaerobic digestion. Even though digesters with low diversity might operate under stable conditions, higher microbial diversity ensures a higher resistance resilience and functional redundancy, which ultimately results in good AD performances [42].

Since hydrolysis is the first step on anaerobic digestion, it can be assumed that these data reflected a higher proteolytic activity of S1 with regard to the other tested sludge samples. Proteolytic bacteria occur in high numbers in anaerobic digesters receiving raw sewage sludge [24]. This would support the fact that S1-SMA was the highest since the other sludge samples were adapted to digest mainly microalgae biomass. More interestingly, S1 was the sludge with lower *Proteobacteria* relative abundance (35% out of the total bacterial number *vs*. 46–51% for the adapted sludge, Fig. 1a). In this manner, it can be highlighted that despite the lower relative abundance, the enzymes secreted by the proteolytic bacteria of S1 were more suitable and/or active for BSA hydrolysis. The SMA using BSA as a substrate was diminished along digestion time. After approximately 7 days of digestion, the SMA attained for BSA was minimal (Fig. 3a) while the one corresponding to cellulose as model substrate slowly increased (Fig. 3b). Thus, it could be concluded that all sludge samples had a higher proteolytic activity than cellulolytic toward the model substrates. One or other prevalent hydrolytic activity strongly depend on the selected inoculum. In principle, protein hydrolysis is slower than the hydrolysis of carbohydrates [43]. Nevertheless, hydrolysis constants and, therefore, methane production is highly dependent on several factors such as microbial community, selected model substrate and digestion temperature. In this manner, a wide range of hydrolytic constant might be found in the literature [44].

As observed for S1 and the adapted sludge samples (S4–S6), the SMA obtained when using cellulose model substrate was also clearly different (Fig. 3b). The only common behavior for all the sludge samples was the lack of methane production during the 2–3 first days of digestion. Similarly to what it was observed in the previous case, S1 exhibited three-fold higher SMA than the adapted sludge samples after 5 days of digestion (0.07 g COD consumed/g VS day for S1 vs ± 0.022 g COD consumed/g VS day for S4–S6). Once again, the higher biodiversity determined in S1 mediated higher SMA for cellulose than the rest of the sludge samples. While in the case of the proteolytic activity, SMA steadily decreased along digestion, in the case of cellulolytic activity, the sludge samples responded differently. Almost barely any activity was observed after 7 days of digestion for S1, while the adapted sludge samples maintained an SMA of 0.021–0.024 along 19 days of digestion regardless of the digestion temperature. This fact resulted in a similar methane yield but lower methane productivity of the adapted sludge samples.

### Microalgae digestion using different anaerobic sludge inocula

**Fig. 4:**
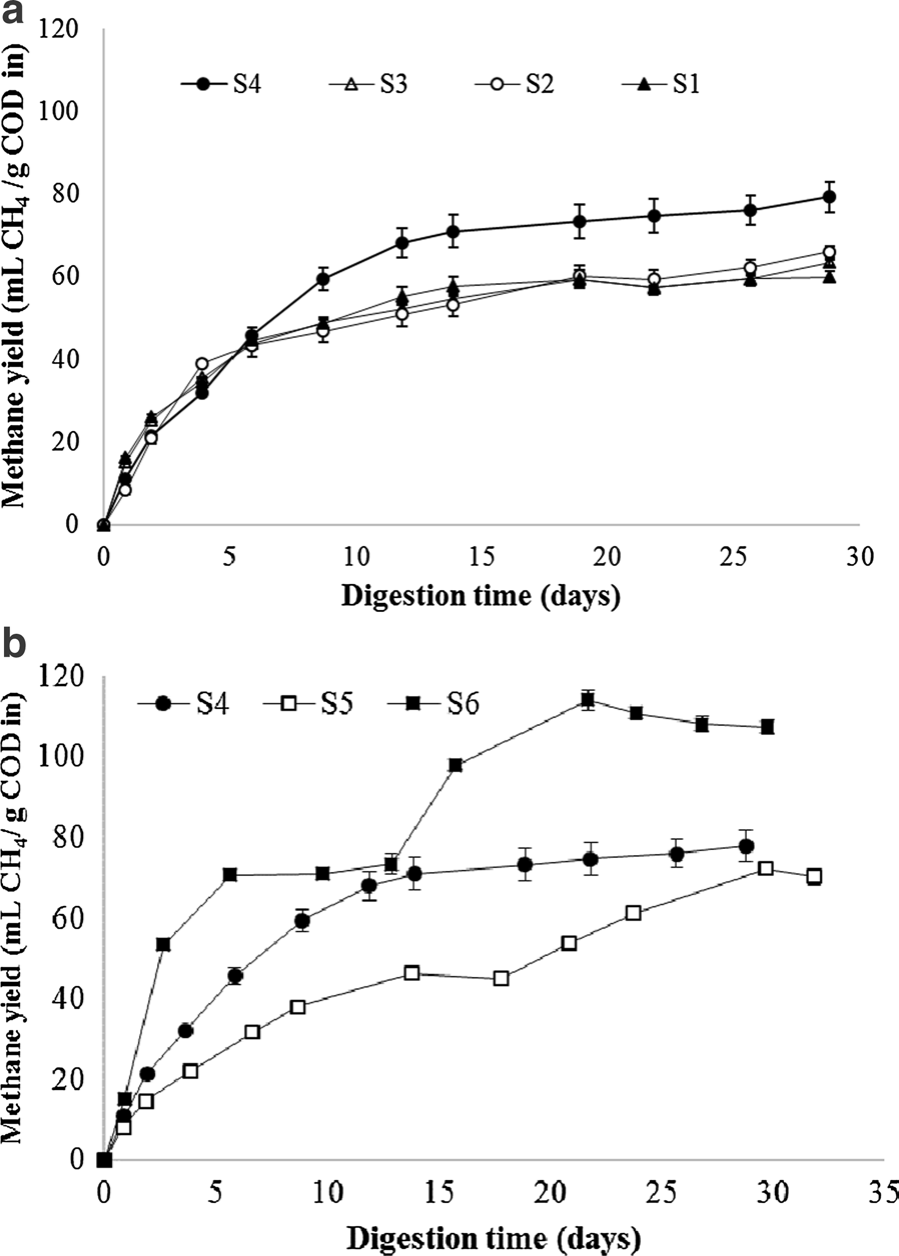
Cumulative methane production achieved by mesophilic sludge samples (**a**) and adapted to microalgae sludge samples (**b**) when digesting *Scenedesmus* sp. biomass

Based on the above-mentioned analysis, it was obvious that differences existed among the inocula used herein. Incubation with different inocula at mesophilic range resulted in similar BMP values for *Scenedesmus* sp. biomass, since no significant differences were observed between the different non-adapted mesophilic inocula (Fig. 4a). S1–S3 supported methane yields of 63.1 ± 3.1 mL CH_4_ g COD in^−1^. Slightly higher values (79.2 ± 3.1 mL CH_4_ g COD in^−1^) were determined for the sludge adapted to digest microalgae biomass (S4). With regard to the sludge samples adapted to digest microalgae biomass at different temperature, methane yields increased concomitantly with digestion temperature. More specifically, the values attained were 63.4 ± 1.5, 79.2 ± 3.1 and 108.2 ± 1.9 mL CH_4_ g COD in^−1^ for psychrophilic, mesophilic and thermophilic digestions, respectively (Fig. 4b). Despite of the differences registered in terms of the anaerobic microbiome and the metabolic activities towards model substrates, no big differences were evidenced among the mesophilic sludge samples. This feature was opposite to what it is described in the literature when testing different inocula sources for the digestion of different biomass. For instance, Gu et al. [45] evaluated different inocula sources on the anaerobic degradation of rice straw and their results showed that digested manures were more active than anaerobic sludge. Similarly, Cordoba et al. [11] also proved that the selection of inocula sources could affect the anaerobic digestion of liquid swine wastewater. Nevertheless, in some cases, the effect of inocula source is only detectable depending on the targeted substrate [46]. In the present study, the only remarkable differences were observed for the sludge samples adapted to digest microalgae biomass at meso- and thermophilic range. In principle, thermophilic digesters are usually operated as close as possible to 50 °C. This temperature, being close to the optimum for enzymatic activity, frequently results in faster reaction rates compared to mesophilic digestion, leading to shorter retention times. Therefore, advantages of thermophilic digestion involve faster hydrolysis and acidogenesis while being more sensitive to ammonia toxicity. Within thermophilic digestion of microalgae in BMP mode, Capson-Tojo et al. [47] digested lipid-extracted *Nannochloropsis gaditana* at mesophilic and thermophilic range. They concluded that mesophilic digestion supported higher anaerobic biodegradability than thermophilic, however, they also observed that this later digestion temperature supported higher COD solubilization. This was attributed to the fact that the used anaerobic sludge was indeed mesophilic for both assays, and thus, the digestion run at thermophilic range was too short to get the anaerobic microorganisms adapted to the thermophilic temperature. As a matter of fact, the investigation presented herein proved that when the inoculum was adapted to digest microalgae at thermophilic conditions, methane yield was the highest of all trials. Overall, adapted sludge samples to the digestion of microalgae have shown to be beneficial for the biodegradation of *Scenedesmus* sp. Digestion conducted at psychrophilic range supported similar methane yields than the sludge samples adapted to digest sewage sludge (Fig. 4). Therefore, the same yield could be achieved at lower energy cost for maintaining the digester temperature. Methane yields of psycro- and mesophilic digestions were quiet similar most likely due to their anaerobic microbiome resemblance (bacterial and archaeal population). The most remarkable difference in methane yields was achieved by the thermophilic consortium, which provided the highest value. Methane yield at thermophilic range was 1.36-fold higher than mesophilic range. The benefits of using thermophilic digestion over mesophilic digestion seems to be specie specific [5]. According to Zamalloa et al. [48], the digestion of *S. obliquus* in thermophilic range increased the methane yield by 24% when compared to the digestion in mesophilic range operating continuous digesters. To the best of the authors’ knowledge, no thermophilic digestion in batch mode has been published for *Scenedesmus* and thus, no comparison could be made. Nevertheless, pretreatments devoted to enhance methane yields of this microalgae strain in BMPs reported similar enhancement values [49]. Thus, this study highlighted the potential of working with highly specific consortia to increase methane yield. Given the differences in the anaerobic microbiomes of the different sludge samples, it can be concluded that the microorganism’s consortium developed in the adapted sludge was linked to the higher methane yields achieved. As hypothesized by Greses et al. [34] the results obtained herein seemed to confirm that the high relative abundance of Firmicutes in the thermophilic sludge (Fig. 2a) compared to the rest of the sludge samples could be the explanation for the higher methane yields achieved with this inocula.

**Fig. 5:**
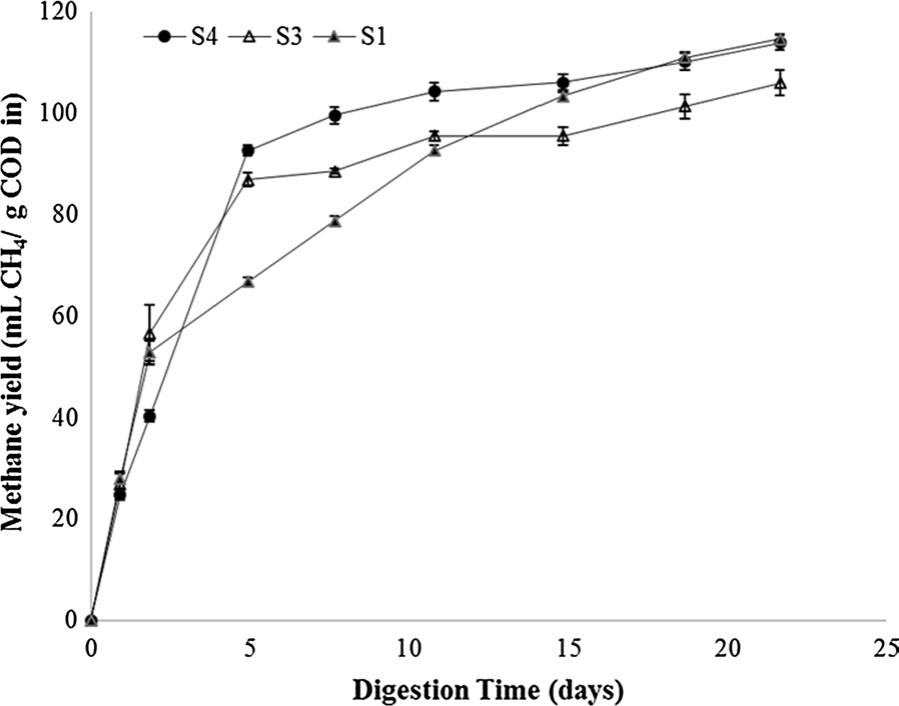
Cumulative methane production achieved by mesophilic sludge samples when digesting *Chlorella sorokiniana* biomass

According to De Vrieze et al. [46] the substrate employed could be of paramount importance when dealing with the effect of different inocula sources. Since *Scenedesmus* sp. is most probably the hardest microalgae to digest [15], a similar approach was conducted with some easier digestible microalgae. The results attained for the digestion of *Chlorella sorokiniana* upon the use of selected inocula was shown in Fig. 5. The main difference was the methane productivity while the final yields were not affected by the inoculum. After 15 days of digestion, methane yields ranged 105–114 mL CH_4_ g COD in^−1^ for the three tested sludge. In this context, the beneficial effect observed on the anaerobic digestion of *Scenedesmus* sp. by the inoculum adapted to digest microalgae (S4) was not evident in the digestion of *C. sorokiniana*. Nevertheless, it cannot be neglected that the productivity was higher for S4 than for the rest of the sludge samples. In this manner, S4 achieved maximum methane yield after 10 days of digestion while S1 required 18 days, despite of the highest specific methanogenic activity registered for this sludge (Fig. 3). When compared to *C. sorokiniana*, the better results obtained for *Scenedesmus* sp. were related to the fact that the microalgae biomass digested by S4–S5 and S6 was mainly composed by *Scenedesmus*, *Dictyosphaerium*, *Coelastrum*, *Micractinium* and *Chlorella*. Most probably, the anaerobic microbiome developed in the anaerobic inocula of the adapted sludge samples was particularly suitable for the digestion of *Scenedesmus* and thus, the positive effect was more evident in that biomass.

## Conclusion

This research demonstrated that despite the differences related to their anaerobic microbiome and SMA towards model substrates, the anaerobic digestion of microalgae biomass was not influenced by different inocula sources adapted to digest sewage sludge. Opposite to that, the sludge samples adapted to digest microalgae biomass exhibited better performances. Mesophilic sludge adapted to the digestion of microalgae consortium mainly composed by *Scenedesmus* showed greater methane yields than adapted to digest sewage sludge. The same could not be concluded with other microalgae biomass (*Chlorella*). Thus, it could be concluded that the anaerobic microbiome was tailored to degrade mainly *Scenedesmus*. With regard to the adapted inocula, psychrophilic digestion displayed lower methane productivity while methane yield was comparable to mesophilic digestion with adapted sludge. Most remarkably was the methane yield achieved by the thermophilic adapted sludge. Even though, a high microbial diversity might play a positive role in maintaining the stability of the system, the anaerobic microbiome of thermophilic digester presented a low diversity but highly efficient for the anaerobic digestion of *Scenedesmus* sp. The relative abundance of Firmicutes, particularly *Clostridia*, and Proteobacteria together with an important abundance of hydrogenotrophic methanogens was highlighted in this inoculum. Linking process engineering to microbial community in AD reactors could bring new insights to pay the way out to a better digester performance and avoid pretreatments by working with a highly specific anaerobic microbiome.
